# Factors Associated with High Prevalence of Intestinal Protozoan Infections among Patients in Sana'a City, Yemen

**DOI:** 10.1371/journal.pone.0022044

**Published:** 2011-07-18

**Authors:** Naelah A. Alyousefi, Mohammed A. K. Mahdy, Rohela Mahmud, Yvonne A. L. Lim

**Affiliations:** 1 Department of Parasitology, Faculty of Medicine, University of Malaya, Kuala Lumpur, Malaysia; 2 Department of Parasitology, Faculty of Medicine, Sana'a University, Sana'a, Yemen; Louisiana State University, United States of America

## Abstract

**Background:**

Intestinal protozoan diseases in Yemen are a significant health problem with prevalence ranging from 18% to 27%. The present study is a cross-sectional study aimed at determining the factors associated with the high prevalence of intestinal protozoan infections among patients seeking health care in Sana'a City, the capital of Yemen.

**Methodology/Principal Findings:**

Stool samples were collected from 503 patients aged between 1 and 80 years old; 219 were males and 284 females. Biodata were collected via pretested standard questionnaire. Faecal samples were processed and examined for (oo)cysts or ova using a wet mount preparation after formal-ether concentration technique. Cryptosporidium oocysts were detected using the Ziehl-Neelsen staining technique. The overall prevalence of intestinal protozoan infections was 30.9%. Infection rates of *Giardia duodenalis*, *Entamoeba histolytica/dispar and Cryptosporidium* were 17.7%, 17.1% and 1%, respectively. Other parasites detected included *Ascaris lumbricoides* (2.4%), *Schistosoma mansoni* (0.3%), *Hymenolepis nana* (1.4%) and *Enterobius vermicularis* (0.4%). Multivariate analysis using forward stepwise logistic regression based on intestinal protozoan infections showed that contact with animals (OR = 1.748, 95% CI = 1.168–2.617) and taking bath less than twice a week (OR = 1.820, 95% CI = 1.192–2.779) were significant risk factors of protozoan infections.

**Conclusions/Significance:**

This present study indicated that intestinal protozoan infections are still a public health problem in Yemen, with *Giardia* and *Entamoeba* infections being most common. Statistical analysis indicated that low personal hygiene and contact with animals were important predictors for intestinal protozoan infections. As highlighted in this study, in order to effectively reduce these infections, a multi-sectoral effort is needed. Preventive measures should include good hygienic practices, good animal husbandry practices, heightened provision of educational health programs, health services in all governorates including rural areas. Furthermore, it is also essential to find radical solutions to the recent water crises in Yemen.

## Introduction

**Figure 1:**
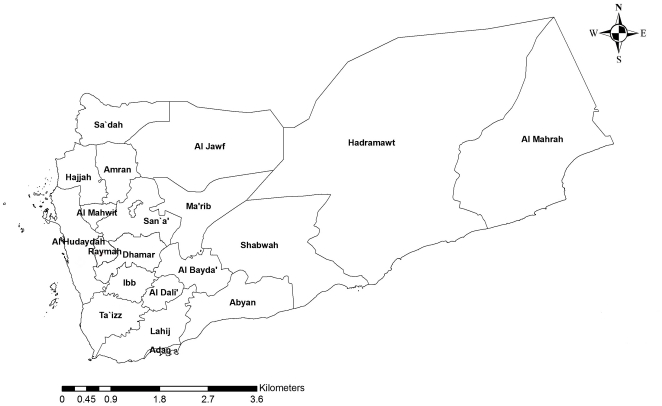
Map of Yemen indicating the location of major cities and governorates.

Yemen is a developing Middle Eastern country located at the southern part of the Arabian Peninsula with a total population of 23 million (Figure 1). The country depends totally on ground water and rain water as a source of water. Recently, the country has fallen into a deep water crisis characterized by very rapid mining of groundwater, extreme water supply shortages in the major cities, and limited access of the population to safe drinking water. WHO reported that only 25% of the population had easy access to safe water [1]. Being one of the poorest countries in the Middle East with a per capita income of approximately USD510, 42% of Yemen's total population is estimated to be under the national poverty line [2]. The poverty ratio is higher in the rural area where 75% of population lives and only 25% is covered with health care services compared to 80% of urban area. This economic imbalance coupled with the current water scarcity have also encouraged or sustained the high prevalence of intestinal protozoan infections in Yemen.

Although intestinal parasitic infections had received attention in Yemen as early as 1950s, most of these efforts were to combat schistosomiasis, unfortunately neglecting the other intestinal parasites [3], [4]. In 1985, a large scale survey by Raja between 1982–1983 involving 37,000 stool specimens showed that 53% of the study population had intestinal parasitic infections with *Trichuris trichiura*, *Ascaris lumbricoides* and *Giardia duodenalis* recording high prevalences [5]. In 2000, Raja and his colleagues carried out another survey in seven villages in the rural areas of Ibb governorate. In this study, the prevalence of *G. duodenalis* and *Entameoba histolytica/dispar* was 18% and 14%, respectively [6]. A recent study carried out on 303 restaurant workers in 58 restaurants in Sana'a City reported high prevalence of *G. duodenalis* (35.3%) and *E. histolytica/dispar* (48.9%) [7]. The infection rates of *G. duodenalis* and *E. histolytica/dispar* were also reported at 18.2% and 52%, respectively, in a similar study conducted on 132 restaurant workers in Almukala City, Hadramowat [8]. The high prevalence of these two intestinal protozoa among restaurant workers is alarming with the possible risk of foodborne outbreak.

Hitherto several studies have focused on parasitic infections among children in Yemen. A cross-sectional study, carried out on 104 children from lowland and highland areas in the south of Yemen showed that, the prevalence of *G. duodenalis* in the lowland and highland areas were 34.6% and 35.1%, respectively. Children living in the lowland areas had higher infection rate of *E. histolytica/dispar* (42.3%) compared to those living in the highland areas (36.8%) [9]. In a hospitalized based study carried out on 9,014 children in the pediatric health center in Sana'a City, the prevalence of *G. duodenalis* and *E. histolytica/dispar* was 16.7% and 11.7%, respectively [10]. A recent study among children in orphanages, reported high prevalence of *G. duodenalis* (26.8%), *E. histolytica/dispar* (13%) and *Cryptosporidium* (24%) [11]. Another study on children was in Hadramowat in 2010 which recorded prevalence of 19.2% for *Giardia* and 16.8% for *E. histolytica/dispar* infections, highlighting that parasitosis reflected the prevalent hygienic problems and their influences on public health of Hadramowat [12].

What was alarming in Yemen was that most studies in different localities and different populations have shown a high prevalence of intestinal protozoan infections, especially with *G. duodenalis* and *E. histolytica/dispar* infections and these rates have not indicated any sustainable reduction since the 1980s. Besides the high rates of infections, not much is known about factors contributing to the high prevalence. Therefore, the present study is a cross-sectional study aimed at determining prevalence and factors associated with the high prevalence of intestinal protozoan infections in Yemen. This study will assist in understanding the mode of transmission as well as to accommodate human health practices, which opposes the transmission of protozoan infections. Indeed, identifying predictors of intestinal protozoan infections is crucial for the effective implementation of control strategies in combating these intestinal protozoan infections.

## Materials and Methods

### Study area and study population

This study was conducted among outpatients at Al Jomhury hospital, Al-Kuwait hospital and Alzahrawy clinical center in Sana'a city, Yemen. Faecal samples were collected from patients referred to the parasitological lab for stool examination. A total of 503 samples were collected. Patients were invited to participate voluntarily after a clear explanation of the objectives of the study was provided and written consent obtained. If the patients were children, written informed consents were obtained from their parents. The study protocol was approved by the research and ethical committee of Faculty of Medicine, University of Malaya, Malaysia (MEC RF. No: 782.9). Permission was obtained from the hospital authorities before the commencement of the study.

### Questionnaire

Prior to sample collection, a brief explanation of the aims of study was given to all patients who voluntarily participated. Information was collected through a pre-tested standard questionnaire which included socio demographic information such as age, gender, education, residence, education level, occupation and monthly income [range from <20000 Ryal Yemeni (<100USD) to >20000 Ryal Yemeni (>100USD)], health practices (i.e., personal awareness of food and water handling beside adoption of health hygiene) and behavioral habits (i.e., washing hands before eating, washing hands after defecation, eating with hands, washing fruits and vegetables and taking bath at least twice a week), health conditions with history of symptoms (e.g., diarrhea, nausea, vomiting and abdominal pain). Environmental conditions such as water supply, provision of sanitation system and existence of animal in households were also included. Age of participants was categorized into two groups that were below 12 years and above 12 years according to a previous study [13].

### Faecal collection and examination

The stool samples were collected from patients in wide mouthed screw-capped containers and labeled. Primary detection of (oo)cysts and ova were made by examination of direct smear, which was prepared from fresh stool. Wet mount preparation after formal ether concentration method was also made to increase the sensitivity of the detection using the light microscopy [14]. *Cryptosporidium* oocysts were detected using modified Ziehl-Neelsen stain technique. Infected cases were treated by medical personnels from the respective hospitals in Yemen. All samples were examined at Al Jomhury hospital parasitological laboratory and later preserved in potassium dichromate solution for further investigations.

### Statistical analysis

Data were analyzed using SPSS programme for windows version 11.5 (SPSS Inc., Chicago.IL, USA). Univariate analyses were used to investigate the association between dependent and independent variables. The significance was defined as *p*<0.05. Those variables that showed significance with *p*<0.05 were used to develop a stepwise forward logistic regression model.

## Results

A total of 503 samples were collected from patients attending different hospitals and health center, in Sana'a City, which were referred to a parasitological laboratory for faecal examination. Of these patients, 219 were males and 284 were females. The age of participants was between 1 and 80 years old. The overall prevalence of parasitic infection was 40.3%. Multiple infections were registered at 11.7%, with 30.9% of protozoan infection. The prevalence of each parasite is indicated in Table 1. *Giardia duodenalis* had the highest infection rate (17.7%) followed by *Entamoeba histolytica/dispar* (17.1%). Other intestinal parasites detected include *Ascaris lumbricoides* (2.4%), *Hymenolepis nana* (1.4%), *Enterobius vermicularis* (0.4%) and *Schistosoma mansoni* (0.3%).

**Table 1:** Prevalence of intestinal parasitic infections according to species (N = 503).

Parasite	No Infected	%
*Giardia duodenalis*	89	**17.7%**
*Entamoeba histolytica/dispar*	86	**17.1%**
*Cryptosporidium*	5	1%
*Ascaris lumbricoides*	12	2.4%
*Schistosoma mansoni*	2	0.3%
*Hymenolepis nana*	7	1.4%
*Enterobius vermicularis*	2	0.4%
**Total**	**203**	**40.3%**

Univariate analysis identified six factors associated with intestinal protozoan infections (Table 2) which include contact with animals (OR = 1.75, 95% CI 1.17–2.62), not washing fruits and vegetables before eating (OR = 1.66, 95% CI 1.060–2.601), drinking untreated water (OR = 1.50, 95% CI 1.01–2.25), taking bath less than twice a week (OR = 1.82, 95% CI 1.19–2.78), watering plants using untreated water (OR = 1.85, 95% CI 1.07–3.21) and working mother (farmers) (OR = 2.26, 95% CI 1.22–4.17). Those living in the rural areas (OR = 1.52, 95% CI 0.99–2.305) and do not practice hand washing (OR = 1.47, 95% CI 0.99–2.17) had higher infection rate. Multivariate analysis using forward stepwise logistic regression confirmed contact with animals (OR = 1.75, 95% CI 1.17–2.62) and taking bath less than twice a week (OR = 1.82, 95% CI 1.19–2.78) as significant risk factors of intestinal protozoan infections.

**Table 2:** Factors associated with protozoan infections among patients seeking health care in Sana'a City.

Variables		Infected%	OR (95%CI)	*p *value
Age (years)	>12≤12	29.634	11.23(0.81–1.85)	0.33
Address	UrbanRural	28.437.6	11.52(0.99–2.31)	0.050
Gender	MaleFemale	29.432.2	11.14(0.78–1.67)	0.50
Income(Yemeni Ryal)	>20000≤20000	31.428.9	10.89(0.55–1.45)	0.63
Family size	<5≥5	27.932.2	11.23(0.81–1.86)	0.34
Education	EducatedNot educated	33.326.9	10.74(0.49–1.09)	0.13
Occupation	WorkingNot working	27. 832.5	10.80(0.53–1.213)	0.29
Sewage disposal	Common drainageOthers	29.638.0	11.459(0.87–2.46)	0.15
Existence of animal	NoYes	27.339.6	11.75(1.17–2.617)	**<0.05** *
Washing hands before eating	YesNo	26.534.6	11.47(0.99–2.169)	0.055
Eating raw vegetables	NoYes	31.631.2	10.98(0.61–1.59)	0.94
Eating fresh fruits	NoYes	28.733	10.82(0.56–1.19)	0.29
Washing fruitsand vegetables	YesNo	29.040.4	11.66(1.06–2.601)	**<0.05**
Drinking water	TreatedNot treated	25.934.5	11.50(1.01–2.25)	**<0.05**
Bathing two times weekly	YesNo	27.741.1	11.82(1.19–2.78)	**<0.05** *
Washing hands after defecation	YesNo	28. 633.7	11.27(0.86–1.873)	0.23
Watering plants	NoYes	29.743.3	11.185(1.07–3.20)	**<0.05**
Diarrhea	NoYes	29.633.72	11.28(0.81–1.80)	0.35
Father occupation	OthersFarmer	35.229.1	11.32 (0.87–2.00)	0.19
Mother occupation	OthersFarmer	28.947.8	12.26(1.22–4.17)	**<0.05**

*Confirmed by logistic regression.

In addition, univariate analysis was also carried out based on single infection with *Giardia* (Table 3) and *E. histolytica/dispar* (Table 4). It was found that drinking untreated water was a significant predictor of giardiasis (OR = 1.73. 95% CI = 1.05–2.86). Unexpectedly, people with income ≤20000 Yemeni Ryal appeared as a protective factor (OR = 0.4, 95% CI = 0.24–0.95) (Table 3). Logistic regression analysis confirmed that drinking untreated water was a significant risk factor of *Giardia* infection in Yemen (OR = 2.09, 95% CI = 1.22–3.61).

**Table 3:** Factors associated with G. duodenalis infection among patients seeking health care in Sana'a City.

Variables		Infected%	OR (95%CI)	*p* value
Age (years)	>12≤12	16.820.1	11.25(0.77–2.05)	0.37
Address	UrbanRural	16.121.8	11.45(0.88–2.39)	0.14
Gender	MaleFemale	15.619.4	11.31(0.82–2.09)	0.27
Income(Yemeni Ryal)	>20000≤20000	19.510.3	10.47(0.24–0.95)	**<0.05**
Family size	<5≥5	17.817.5	11.02(0.62–1.68)	0.94
Education	EducatedNot educated	19.115.2	10.76(0.47–1.23)	2.26
Occupation	WorkingNot working	18.815.2	10.77(0.46–1.29)	0.33
Sewage disposal	common drainageOthers	17.121.1	11.31(0.70–2.44)	0.40
Existence of animal	NoYes	17.718.0	11.10(0.67–1.81̀1	0.70
Washing hands before eating	YesNo	18.017.7	10.98(0.61–1.56)	0.92
Eating raw vegetables	NoYes	15.818.2	11.18(0.64–2.17)	0.59
Eating fresh fruits	NoYes	16.718.9	10.86(0.54–1.36)	0.52
Washing fruitsand vegetables	YesNo	17.420.2	10.86(0.54–1.37)	0.51
Drinking water	TreatedNot treated	13.220.8	11.73(1.05–2.86	**<0.05** *
Bathing two times weekly	YesNo	16.020.8	11.60(0.97–2.64)	0.06
Washing hands after defecation	YesNo	16.120.3	11.33(0.83–2.12)	0.20
Watering plants	NoYes	17.023.3	11.49(0.78–2.84)	0.23
Diarrhea	NoYes	15.921.7	11.47(0.92–2.35)	0.11
Father occupation	OthersFarmer	19.316.7	11.19(0.72–1.98)	0.49
Mother occupation	OthersFarmer	16.326.5	11.78(0.88–3.61)	0.10

*Confirmed by logistic regression.

**Table 4:** Factors associated with E. histolytica/dispar infection among patients seeking health care in Sana'a City.

Variables		Infected%	OR (95%CI)	p value
Age (years)	>12≤12	15.518.8	11.17(0.71–1.93)	0.54
Address	UrbanRural	15.023.3	11.72(1.05–2.82)	**<0.05**
Gender	MaleFemale	17.916.6	10.91(0.57–1.45)	0.71
Income(Yemeni Ryal)	>20000≤20000	17.517.0	11.04(0.58–1.86)	0.91
Family size	<5≥5	19.312.3	11.69(0.98–2.93)	0.06
Education	EducatedNot educated	16.317.8	11.07(0.67–1.71)	0.26
Occupation	WorkingNot working	17.916.5	10.90(0.55–1.50)	0.33
Sewage disposal	Common drainageOthers	15.626.8	11.97(1.10–3.55)	**<0.05**
Existence of animal	NoYes	12.528.2	12.75(1.71–4.43)	**<0.05** *
Washing hands before eating	YesNo	11.421.9	12.19(1.31–3.64)	**<0.05** *
Eating raw vegetables	YesNo	16.920.0	10.81(0.46–1.43)	0.47
Eating fresh fruits	NoYes	16.718.9	10.86(0.54–1.37)	0.52
Washing fruitsand vegetables	YesNo	15.624.0	11.71(1.01–2.89)	**<0.05**
Drinking water	TreatedNot treated	13.219.9	11.63(0.98–2.69)	0.06
Bathing two times weekly	YesNo	14.924.2	11.82(1.10–2.99)	**<0.05**
Washing hands after defecation	YesNo	15.218.8	11.29(0.80–2.09)	0.29
Watering plants	NoYes	15.628.3	12.13(1.15–3.95)	**<0.05**
Diarrhea	NoYes	15.718	10.85(0.51–1.40)	0.52
Father occupation	OthersFarmer	15.222.8	12.33(1.18–4.59)	**<0.05**
Mother occupation	OthersFarmer	15.830.4	11.97(1.10–3.55)	**<0.05**

*Confirmed by logistic regression.

*E. histolytica/dispar* infection was significantly associated with contact with animals (OR = 2.75, 95% CI = 1.71–4.43), watering activity (OR = 2.13, 95% CI = 1.15–3.95), taking bath less than twice a week (OR = 1.82, 95% CI = 1.10–2.99), not washing hands before eating (OR = 2.19, 95% CI = 1.31–3.64), living in rural areas (OR = 1.72, 95% CI = 1.05– 2.82) and the absence of common drainage (OR = 1.97, 95% CI = 1.09–3.54) (Table 4). Logistic regression confirmed that not washing hands before eating (OR = 1.98, 95% CI = 1.15–3.41) and contact with animals as significant predictors of *Entamoeba* infection (OR = 3.09, 95% CI = 1.88–5.06).

## Discussion

The current findings indicated that the prevalence of intestinal protozoan infections was 30.9% based on a single stool sampling. This infection rate was low compared to a previous study carried out on 37,000 outpatients in which the prevalence of intestinal protozoa was 53% [5]. In comparison to other Mediterranean countries, the infection rate with intestinal protozoa in this study is higher than previous reports from Saudi Arabia among patients seeking health care (27.8%–32.2%) [15], [16], Iran (19.9%) [17], [18] and Oman (18%) [19], however lower compared to the prevalence in Pakistan (52%) [20]. The most dominant protozoa in this study were *G. duodenalis* and *E. histolytica/dispar*, which were rated at 17.7% and 17.1%, respectively. This finding was comparable to the previous studies carried out in Yemen [6], [10], except studies among restaurant workers [7], [8]. These differences could be attributed to the differences in the study subjects and study areas. Besides that, different diagnostic methods used from one study to another should also be considered as a possible reason behind the disparity in the infection rates [21], [22]. This study showed a significant association between low personal hygiene practices and behavior with intestinal protozoan infections. Logistic regression analysis indicated that people who did not take their bath at least twice a week were at 2-fold higher risk of getting infection with intestinal protozoa. Undoubtedly, the current water crisis contributed in some ways to the low hygienic practices in Yemen, leading to the increase of intestinal parasitic diseases.

Yemen depends totally on ground water, which is dropping by 20–65 feet a year as reported by the World Bank [23]. Furthermore, the Carnegie Endowment for International Peace noted that 19 of Yemen's 21 main aquifers were not being replenished because of lower rainfall [24]. The impact of water quantity on the health status has been well documented since plentiful and accessible supplies of water do encourage better hygiene. Two review studies which covered 84 studies in 28 countries have concluded that quantity of water available has more impact on endemic diarrhea cases in developing countries than water purity [25], [26]. Another study carried out in Nicaragua found that children from homes with insufficient water supply had 34% higher infection rate of diarrhea [27]. Water availability may also affect the frequency of hand washing as it has been stated that a mother needs 20 liters of water to wash her hands after using the latrine, changing a nappy, before preparing food, eating, giving food to the infant and handling of cooking or drinking utensils [28].

In the present study, logistic regression analysis also showed that those people who came in contact with animals were at significant risk in acquiring protozoan infections. This association implicated animals as a significant source of protozoan infection in Yemen. However, this postulation should be confirmed by further studies which incorporate molecular tools. It is common for the rural communities in Yemen to keep animals such as cattle, goats and donkeys in the ground floor of the same house. Evidence of zoonotic transmission of some intestinal protozoa, especially *Giardia* and *Cryptosporidium* have been provided by several studies via molecular data analysis [29], [30].

With regards to *Giardia* infection, it has been shown to be significantly associated with drinking untreated water. In Yemen, ground water is the main source of drinking water. Given that most of the homes are without a proper sanitary system, the possibility of faecal contamination is high via ground seepage [31]. Furthermore, it was noted that people in rural areas are dependent on dams besides wells as drinking water resources. Dam water is a collection of rainwater, which is exposed to high pollution, especially during the rainy season due to soil runoff contaminated with parasite (oo)cysts and ova from animal and human faeces. Previously, the using of well water has been identified as significant predictors of *E. histolytica* and *Giardia* infections in Saudi Arabia. Comparatively, those who use desalinated water have the lowest degree of exposure to the risk of infection [32]. Contamination of drinking water with *Giardia* cyst during transporting and storing of drinking water are highly possible as rural people in Yemen use containers to transfer water from the dams or wells to the houses where water is stored to be used for drinking and cooking. Faecal contamination of drinking water between the source and the point of use is well known [33] and improving household water management has been promoted as low cost health intervention to combat waterborne infections [34].

As for *E. histolytica/dispar* infection, the present findings showed that those who do not practice proper hand washing before eating was at two fold higher risk of acquiring *E. histolytica/dispar* infection. The major role of contaminated hands in the faecal-oral transmission of diseases has been well documented in developing countries and washing hands before eating or after defecation has been considered as a secondary barrier. In Indonesia, it has been reported that people who never or sometimes wash hands had a four times higher risk of getting severe diarrhea [35]. In Nepal, the practice of hand washing had a strong correlation with the prevalence of parasitic infection [36]. Another case-control study in the same country indicated that people who never used soap for washing hands were at 30 times higher risk of typhoid [37]. In addition, not washing hands has been reported to be significantly associated with diarrhea in Malaysia [38] and Myanmar [39]. Intervention trials and case-control studies, conducted in Bangladesh, have also indicated that not washing hands was a significant risk for diarrhea [40], [41], [42].

In addition, animal contact was also identified as a significant factor associated with *Entamoeba* infection. Although it is still unclear whether *Entamoeba* infection is zoonotic or not, this parasite has been isolated from animals. In Ethiopia, a study on baboon and Cercopithecus (old world monkey) found that the prevalence of *E. histolytica* was 24.4% [43]. Another study carried out in Ethiopia found that cockroaches serve as carriers of human intestinal parasites [44]. In Uganda, *E. histolytica* and *Giardia* have been detected in monkeys [45]. Similarly, in the Philippines, *E. histolytica* and *E. dispar* were detected among captive macaques in a primate facility. In the same study, using PCR, they found that 23 *E. histolytica* isolates were identical to human *E. histolytica* [46] highlighting a possibility of zoonotic transmission.

In conclusion, the present study showed high prevalence of intestinal protozoan infections with *E. histolytica/dispar* and *G. duodenalis* being the most predominant protozoa among patients seeking health care in Yemen. Low personal hygiene practices such as not washing hands before eating and the frequency of bathing, besides water contamination seemed to play major roles in the high transmission of intestinal protozoa. Therefore, these factors should be given due consideration when implementing any interventions to combat these intestinal protozoan diseases. Although animals are still a possible source of human infection with protozoa as shown in this study, this postulation warrant further studies especially those that utilize advanced molecular techniques. Genotyping *E. histolytica/dispar* and *G. duodenalis* from human, animals and water resources are highly recommended to understand the actual dynamics of transmission of these protozoa in Yemen. Likewise, the authority must take into consideration the development of health awareness among the community through active encouragement of individuals in adopting hygienic behaviors via audio, visual and curriculum programs. Radical solution to water scarcity is also an important requirement to combat the proliferation of these infections in Yemen.
